# Microglial ASD-related genes are involved in oligodendrocyte differentiation

**DOI:** 10.1038/s41598-021-97257-9

**Published:** 2021-09-08

**Authors:** Yuta Takanezawa, Shogo Tanabe, Daiki Kato, Rie Ozeki, Masayo Komoda, Tatsunori Suzuki, Hiroko Baba, Rieko Muramatsu

**Affiliations:** 1 Department of Molecular Pharmacology, National Institute of Neuroscience, National Center of Neurology and Psychiatry, Kodaira, Tokyo 187-8502 Japan; 2 Department of Molecular Neurobiology, Tokyo University of Pharmacy and Life Sciences, Hachioji, Tokyo 192-0392 Japan; 3 Department of Medical and Life Science, Faculty of Pharmaceutical Sciences, Tokyo University of Science, Noda, Chiba 278-8510 Japan; 4 Department of Pharmacy, Faculty of Pharmaceutical Sciences, Tokyo University of Science, Noda, Chiba Japan

**Keywords:** Cellular neuroscience, Glial biology

## Abstract

Autism spectrum disorders (ASD) are associated with mutations of chromodomain-helicase DNA-binding protein 8 (Chd8) and tuberous sclerosis complex 2 (Tsc2). Although these ASD-related genes are detected in glial cells such as microglia, the effect of Chd8 or Tsc2 deficiency on microglial functions and microglia-mediated brain development remains unclear. In this study, we investigated the role of microglial Chd8 and Tsc2 in cytokine expression, phagocytosis activity, and neuro/gliogenesis from neural stem cells (NSCs) in vitro. Chd8 or Tsc2 knockdown in microglia reduced insulin-like growth factor-1(Igf1) expression under lipopolysaccharide (LPS) stimulation. In addition, phagocytosis activity was inhibited by Tsc2 deficiency, microglia-mediated oligodendrocyte development was inhibited, in particular, the differentiation of oligodendrocyte precursor cells to oligodendrocytes was prevented by Chd8 or Tsc2 deficiency. These results suggest that ASD-related gene expression in microglia is involved in oligodendrocyte differentiation, which may contribute to the white matter pathology relating to ASD.

## Introduction

Autism spectrum disorders (ASDs) are highly inherent neurodevelopmental disorders characterized by repetitive behaviors, restricted interest, and deficits in social interaction^1^. It has been reported that a large number of neurons, excessive synapses, and myelin abnormalities are observed in the brain of patients with ASD, indicating that developmental anatomical abnormalities are considered to be associated with ASD pathology^2–4^.

ASD is known to be closely associated with genetic variation. Recent studies have identified several genes that are frequently mutated in human ASD and have the potential to regulate the expression of other ASD-risk genes. For example, chromodomain-helicase DNA-binding protein 8 (Chd8), which is an ATP-dependent chromatin remodeling protein that controls epigenetic and transcript regulation^5^, represents one of the most high-risk susceptibility genes in ASD^5,6^. Missense, nonsense, and frameshift mutations have all been found in Chd8 alleles, and several missense mutations in Chd8 confer loss-of-function^7^. This is supported by the fact that Chd8-haploinsufficient mice showed behavioral abnormalities associated with ASD. In addition, Chd8 deficiency in oligodendrocytes leads to impaired oligodendrocyte development and hypomyelination^8,9^. Other genes related to ASD, such as Tsc2 (also known as Tuberin), which is a negative regulator of the mammalian target of rapamycin (mTOR) signaling by dephosphorylating Rheb, a G-protein which activates mTOR complex 1 (mTORC1)^10^. Tuberous sclerosis complex (TSC) is a genetic disease characterized by benign tumors, epilepsy, ASD, and loss of function of Tsc2 due to missense mutations in TSC patients^11^. Tsc2 haplodeficient mice also showed neurological deficits, including ASD-like behaviors, and Tsc2 conditional knockout mice showed hypomyelination in oligodendrocyte lineage cells^12,13^. Thus, loss of function of both Chd8 and Tsc2 are considered to be associated with ASD pathology and myelin abnormalities. Although the recent findings about the function of ASD-related genes have been mainly interpreted by neuronal changes, the role of ASD-related genes in glial cells that also express Chd8 and Tsc2 remains unclear.

Microglia are central nervous system (CNS) resident immune cells that play crucial roles in CNS homeostasis^14^. Microglia also contribute to brain development e.g., development of neurons and oligodendrocytes from the subventricular zone^15^, myelin formation in the neonatal brain^16^, and regulation of the number of neural precursor cells (NPCs) by promoting cell proliferation and/or engulfing cells^17,18^. In addition, microglia contribute to myelin development through phagocytosis of myelin sheaths and by interacting with oligodendrocyte lineage cells^19–23^. Recent studies demonstrated that activated microglia are observed in ASD patients, and microglia are associated with ASD pathogenesis and that the suppression of microglia activation attenuates ASD-like behaviors induced by valproate treatment^24–26^. Although microglia are involved in ASD pathogenesis and myelin abnormalities are observed in ASD patients, it remains unclear whether microglial dysfunction caused by the deficiency of ASD-related genes is associated with myelin pathology in ASD. These observations prompted us to investigate whether ASD-related genes in microglia are involved in neural development associated with ASD^27,28^.

In this study, we investigated the role of Chd8 and Tsc2 in microglial cells for cytokine expression, phagocytic activity, neuro/gliogenesis. Silencing Chd8 or Tsc2 expression in microglia altered insulin-like growth factor-1 (Igf1) mRNA expression in microglia following lipopolysaccharide (LPS) stimulation. Additionally, Tsc2 deficient microglia showed reduced phagocytic activity. NSCs co-cultured with microglia promoted oligodendrocyte development; however, Chd8 or Tsc2 knockdown in microglia prevented this microglia-mediated increased in oligodendrocyte differentiation. On the other hand, Chd8 or Tsc2 deficiency in microglia did not affect the differentiation of neurons and astrocytes from NSCs. Mice with Chd8 and Tsc2 knockdown in microglia show a reduced number of differentiating oligodendrocytes in the carpus callosum, anterior commissure, and striatum. These results indicate that Chd8 or Tsc2 in microglia may contribute to white matter pathology in ASD.

## Results

### Chd8 or Tsc2 was effectively down-regulated by siRNA treatment in primary microglia

**Figure 1:**
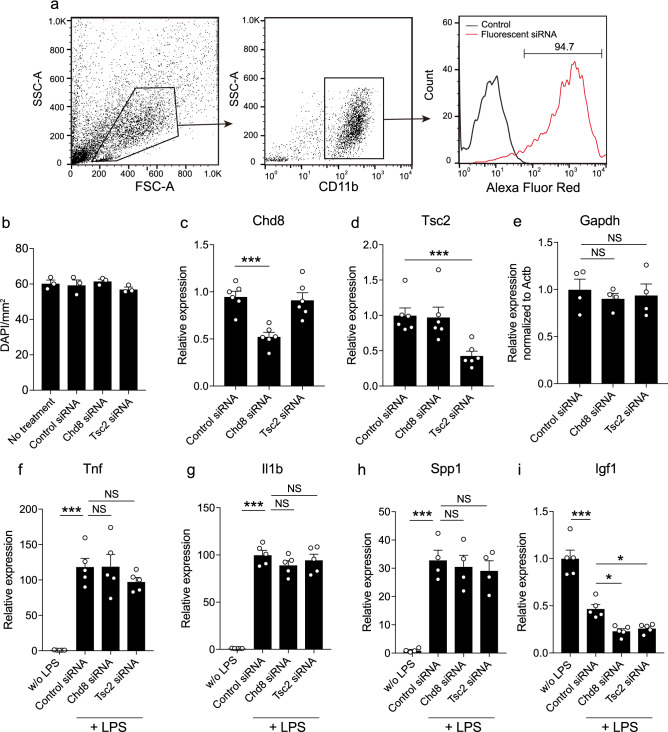
siRNA transduction reduced the expression of ASD-related genes in primary microglia. (**a**) Representative images show the gating strategy of flow cytometry analysis. Primary microglia were transduced red fluorescent siRNA and conducted flow cytometry analysis to confirm the siRNA transduction efficiency after 3 days of transduction. 94.7% of microglia represent red fluorescence. (**b**) A representative graph showing the number of microglia after 3 days of siRNA treatment. Primary microglia were stained with DAPI and observed under fluorescent microscopy (n = 3, biologically independent experiments, F (3.8) = 1.005, df = 8, *p* = 0.4390 for main effect of group assessed by one-way ANOVA). (**c**,**d**) Chd8 (**c**) and Tsc2 (**d**) mRNA expression levels were analyzed by quantitative RT-PCR in primary microglia treated with control siRNA, Chd8 siRNA and Tsc2 siRNA. Expression levels of Chd8 and Tsc2 were normalized to that of Gapdh (n = 6, *p* < 0.001 assessed by the one-way ANOVA, followed by Tukey–Kramer tests). (**e**) Gapdh expression levels were analyzed by quantitative RT-PCR by normalizing to Actb (n = 4, biologically independent experiments, F (2, 9) = 0.2393, df = 9, *p* = 0.7920 for main effect of group, *p* = 0.7789 for control siRNA vs. Chd8 siRNA, *p* = 0.9012 for control siRNA vs. Tsc2 siRNA assessed by the one-way ANOVA, followed by Tukey–Kramer tests). (**f**–**i**) mRNA expression levels of Tnf (**f**; n = 5, biologically independent experiments, F (3,16) = 27.19, df = 16, *p* < 0.001 for main effect of group, *p* < 0.001 for w/o LPS vs. control siRNA, *p* > 0.9999 for control siRNA vs. Chd8 siRNA, *p* = 0.5328 for control siRNA vs Tsc2 siRNA), Il1b (**g**; n = 5, biologically independent experiments, F (3,16) = 108, df = 16, *p* < 0.001 for main effect of group, *p* < 0.001 for w/o LPS vs. control siRNA, *p* = 0.3573 for control siRNA vs. Chd8 siRNA, *p* = 0.8292 for control siRNA vs. Tsc2 siRNA), Spp1 (**h**; n = 4, biologically independent experiments, F (3,12) = 22.36, df = 12, *p* < 0.001 for main effect of group, *p* < 0.001 for w/o LPS vs. control siRNA, *p* = 0.9558 for control siRNA vs. Chd8 siRNA, *p* = 0.9880 for control siRNA vs. Tsc2 siRNA), and Igf1 (**i**; n = 5, biologically independent experiments, F (3,16) = 47.97, df = 16, *p* < 0.001 for main effect of group, *p* < 0.001 for w/o LPS vs. control siRNA, *p* = 0.0232 for control siRNA vs. Chd8 siRNA, *p* = 0.0491 for control siRNA vs. Tsc2 siRNA) in microglia treated with control siRNA, Chd8 siRNA and Tsc2 siRNA were analyzed with quantitative RT-PCR. Statistical analysis was performed by the one-way ANOVA, followed by Tukey–Kramer tests. NS: not significant. *p < 0.05, ***p < 0.001. Error bars represent mean ± SEM.

To examine the role of ASD-related genes in microglia, we established gene knockdown experiments in primary microglia by siRNA treatment. Initially, we transduced fluorescent-labeled siRNA to primary microglia to determine the efficiency of siRNA transduction and measured the ratio of fluorescent-labeled CD11b^+^ microglia by flow cytometry analysis. The analyses revealed that 94.7% of CD11b^+^ microglia exhibited fluorescence, suggesting that siRNA was effectively transduced to primary microglia (Fig. 1a). The number of microglia was not affected by siRNA treatment, indicating that the treatment was not toxic for primary microglia (Fig. 1b). To down-regulate ASD-related genes in microglia, we transduced control siRNA, Chd8 siRNA or Tsc2 siRNA and examined the expression levels by quantitative RT-PCR after 3 days of transduction. Chd8 siRNA and Tsc2 siRNA effectively down-regulated Chd8 and Tsc2 expression in primary microglia, respectively (Fig. 1c,d). Notably, Gapdh was not affected by Chd8 or Tsc2 siRNA treatment (Fig. 1e). To examine whether Chd8 or Tsc2 deficiency in microglia affects cytokine expression levels, we performed quantitative RT-PCR in microglia after stimulating them with LPS for 6 h^29^. LPS stimulation strongly induced the expression of genes encoding tumor necrosis factor (Tnf), Il1b, Spp1, and reduced by half the expression of Igf1. Although Tnf, Il1b, Spp1 expression were not affected by siRNA treatment, Igf1 expression was reduced in both the Chd8 and Tsc2 siRNA treatment groups compared with the control siRNA treatment group (Fig. 1f–i). These results shows that deficiency in Chd8 or Tsc2 in microglia affects expression of Igf1, but not major pro-inflammatory cytokines.

### Knockdown of Tsc2 affected phagocytic activity

**Figure 2:**
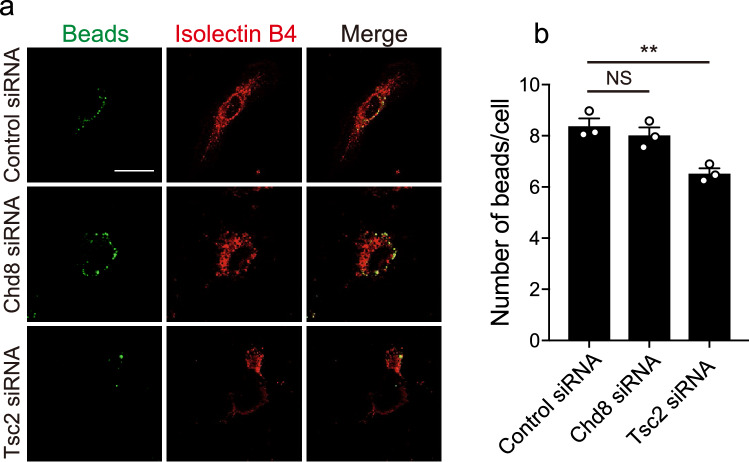
The deficiency in the expression of Tsc2 affects phagocytosis activity in primary microglia. (**a**) Primary microglia transduced siRNA were treated with fluorescent beads (green) to assess phagocytosis. After 24 h, microglia were stained with isolectin B4 (red). Scale bar: 20 μm. (**b**) A graph showing the number of beads in isolectin B4^+^ microglia (n = 3, biologically independent experiments, F (2, 6) = 13.63, df = 6, *p* = 0.0059 for main effect of group, *p* = 0.6335 for control siRNA vs. Chd8 siRNA, *p* = 0.0064 for control siRNA vs. Tsc2 siRNA assessed by one-way ANOVA, followed by Tukey–Kramer tests). *NS* not significant, **p < 0.01. Error bars represent mean ± SEM.

Microglia are involved in brain development by engulfing excessive neurons or synapses during brain development^17,30^. To examine whether the deficiency of Chd8 and Tsc2 in microglia affects phagocytosis activity, we performed phagocytosis assays using fluorescent-labeled latex beads. Quantification of the number of fluorescently-labeled beads in Isolectin B4-labeled microglia indicated a low number of fluorescent-labeled beads in Tsc2 siRNA-treated microglia compared with control (Fig. 2a,b), whereas microglia treated with Chd8 siRNA did not change. These results indicate that Tsc2, but not Chd8, plays a role in phagocytosis activity in primary microglia.

### Deficiency of Chd8 or Tsc2 in microglia led to the impairment of oligodendrocyte development

**Figure 3:**
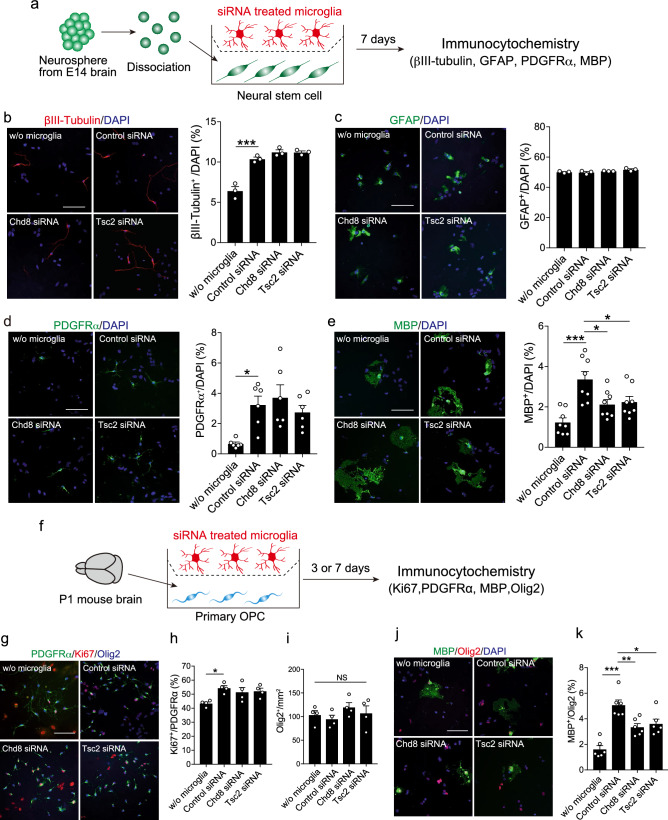
Microglia defecting the Chd8 or Tsc2 expression have impairment in promoting the development of oligodendrocytes. (**a**) NSCs were prepared by the dissociation of neurospheres obtained from E14 embryonic murine brain, and co-cultured with siRNA-treated microglia across the trans-wells. After 7 days, immunocytochemistry for βIII-tubulin, GFAP, PDGFRα, MBP were performed to assess the efficiency of differentiation. (**b**–**e**) Left images show immunocytochemical staining for βIII-tubulin (**b**; n = 3, biologically independent experiments, F (3, 8) = 45.63, df = 8, *p* < 0.001 for main effect of group, *p* < 0.001 for w/o microglia vs. control siRNA), GFAP (**c**; n = 3, from biologically independent experiments, F (3, 8) = 3.262, df = 8, *p* = 0.0805 for main effect of group, *p* = 0.9880 for w/o microglia vs. control siRNA), PDGFRα (**d**; n = 6, biologically independent experiments, F (3, 20) = 5.771, df = 20, *p* = 0.0052 for main effect of group, *p* = 0.0193 for w/o microglia vs. control siRNA), MBP (**e**; n = 8, biologically independent experiments, F (3. 28) = 9.449, df = 28, *p* = 0.0002 for main effect of group, *p* = 0.0217 for control siRNA vs. Chd8 siRNA, *p* = 0.0447 for control siRNA vs. Tsc2 siRNA). Counterstaining was performed with DAPI. Scale bar: 100 μm. Right graphs show the percentage of each marker’s positive cells in DAPI^+^ cells. Statistical analysis was performed by the one-way ANOVA, followed by Tukey–Kramer tests. (**f**) Primary OPCs were co-cultured with siRNA-treated microglia for 3 or 7 days. (**g**) Representative images show the immunocytochemistry of OPCs for PDGFRα (green), Ki67 (red), and Olig2 (blue). Scale bar: 100 μm. (**h**) A graph showing the percentage of Ki67 positive cells in PDGFRα Olig2 double-positive cells (n = 4, biologically independent experiments, F (3, 12) = 4.602, df = 12, *p* = 0.0230 for main effect of group, *p* = 0.0209 for w/o microglia vs. control siRNA, assessed by the one-way ANOVA, followed by Tukey–Kramer tests). (**i**) Graph showing the number of Olig2^+^ cells per mm^2^ (n = 4, biologically independent experiments, F (3, 12) = 0.8705, df = 12, p = 0.4832 for main effect of group, assessed by one-way ANOVA test). (**j**) NSCs prepared from the neurosphere were co-cultured with microglia treated with siRNA for 7 days, and immunostained with MBP (green) and Olig2 (red) antibodies. Counterstaining was performed with DAPI (blue). Scale bar: 100 μm. (**k**) A graph showing the percentage of MBP positive cells in Olig2 positive cells (n = 6, biologically independent experiments, F (3, 20) = 18.1, df = 20, *p* < 0.001 for main effect of group, *p* = 0.0077 for control siRNA vs. Chd8 siRNA, *p* = 0.0253 for control siRNA vs. Tsc2 siRNA assessed by one-way ANOVA, followed by Tukey–Kramer tests). *p < 0.05, **p < 0.01, ***p < 0.001. Error bars represent mean ± SEM.

Microglia promote the differentiation of neurons and oligodendrocytes from NSCs during brain development^15^. Next, we examined whether deficiency of Chd8 or Tsc2 affects the microglia-mediated differentiation of neurons and oligodendrocytes. NSCs were prepared by dissociating neurospheres, and co-cocultured with siRNA-treated microglia across the transwell membranes (Fig. 3a). After 7 days, we counted the number of βIII-Tubulin (Tuj1)^+^ neurons, glial fibrillary acidic protein (GFAP)^+^ astrocytes, platelet-derived growth factor receptor α (PDGFRα)^+^ oligodendrocyte precursor cells (OPC), and myelin basic protein (MBP)^+^ oligodendrocytes by immunocytochemistry. The number of neurons, OPC, and oligodendrocytes, but not astrocytes, significantly increased in the presence of microglia (Fig. 3b–e) consistent with the previous reports^15^. In addition, the number of MBP^+^ oligodendrocytes significantly decreased in the presence of Chd8 or Tsc2 siRNA-treated microglia compared with control siRNA-treated microglia (Fig. 3e). No change was detected in the number of neurons and OPC in the presence of Chd8 and Tsc2 siRNA-treated microglia (Fig. 3b,d). These results suggest that microglia require Chd8 or Tsc2 to promote the oligodendrocyte differentiation.

As OPCs proliferate and differentiate into oligodendrocytes^31^, we asked whether Chd8 or Tsc2 in microglia is involved in OPC proliferation. To this end, we co-cultured OPCs with microglia treated with siRNA and performed immunocytochemistry for Ki67 (proliferation marker), PDGFRα, and oligodendrocyte transcription factor 2 (Olig2) after 3 days (Fig. 3f). We counted the number of Ki67^+^ proliferating cells in PDGFRα and Olig2 double-positive OPCs, and we found that Chd8 or Tsc2 deficiency in microglia did not affect the percentage of Ki67^+^/PDGFRα^+^ Olig2^+^ proliferating OPCs (Fig. 3g,h). There was no significant difference about total number of Olig2^+^ cells between the groups (Fig. 3i). To determine whether Chd8 or Tsc2 deficiency in microglia affects oligodendrocyte differentiation, we co-cultured siRNA-treated microglia and NSCs for 7 days and counted the number of MBP^+^ oligodendrocytes to Olig2^+^ oligodendrocyte-lineage cells. The percentage of MBP^+^/Olig2^+^ cells was lower in the presence of Chd8 or Tsc2 siRNA-treated microglia than in control siRNA-treated microglia (Fig. 3j,k). These results suggest that microglia promote the differentiation from OPC to oligodendrocyte in a Chd8- or Tsc2-dependent manner, with no effect on OPC proliferation.

### Chd8 or Tsc2 knockdown by shRNA in microglia reduced oligodendrocyte differentiation

**Figure 4:**
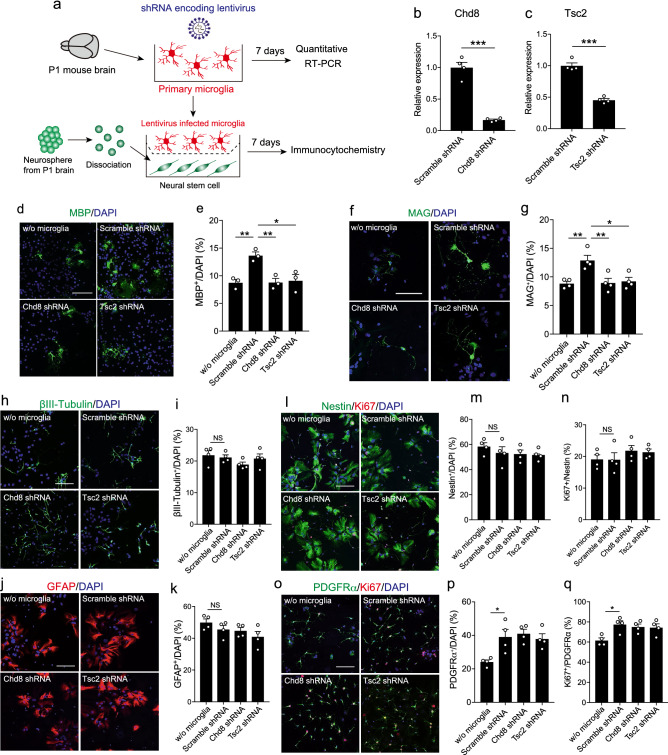
Chd8 or Tsc2 deficiency by shRNA treatment reduced microglia-mediated oligodendrocyte development. (**a**) Primary microglia were infected with lentivirus encoding shRNA against Chd8 or Tsc2. After 7 days, Chd8 and Tsc2 expression levels were examined by quantitative RT-PCR. Infected microglia were co-cultured with NSCs prepared from the P1 mouse SVZ for 7 days. (**b**) Graph showing mRNA expression level of Chd8 in microglia infected with lentivirus (n = 4, biologically independent experiments, F = 36.1, df = 3, *p* = 0.0017 assessed by Welch’s *t*-test). (**c**) mRNA expression of Tsc2 in microglia infected with lentivirus encoding Tsc2 shRNA (n = 4, biologically independent experiments, F = 3.244, df = 3, *p* < 0.001 assessed by Student’s *t*-test). (**d**) Representative images show immunocytochemistry for MBP (green) and DAPI (blue). Scale bar: 100 μm. (**e**) Graph shows the percentage of MBP^+^ cells in DAPI^+^ cells (n = 3, biologically independent experiments, F (3, 8) = 9.367, df = 8, *p* = 0.0054 for main effect of group, *p* = 0.0093 for w/o microglia vs. scramble shRNA, *p* = 0.0098 for scramble shRNA vs. Chd8 shRNA, *p* = 0.0144 for scramble shRNA vs. Tsc2 shRNA assessed by one-way ANOVA, followed by Tukey–Kramer tests). (**f**) Representative images show immunocytochemistry for MAG (green) and DAPI (blue). Scale bar: 100 μm. (**g**) Graph shows the percentage of MAG^+^ cells in DAPI^+^ cells (n = 4, biologically independent experiments, F (3,12) = 7.844, df = 12, *p* = 0.0037 for main effect of group, *p* = 0.0067 for w/o microglia vs. scramble shRNA, *p* = 0.0082 for scramble shRNA vs. Chd8 shRNA, *p* = 0.0134 for scramble shRNA vs. Tsc2 shRNA assessed by one-way ANOVA, followed by Tukey–Kramer tests). (**h**) Representative images show immunocytochemistry for βIII-tubulin (green) and DAPI (blue). Scale bar: 100 μm. (**i**) Graph shows the percentage of βIII-tubulin^+^ cells in DAPI^+^ cells (n = 4, biologically independent experiments, F (3,12) = 1.251, df = 12, *p* = 0.3348 for main effect of group, assessed by one-way ANOVA). (**j**) Representative images show immunocytochemistry for GFAP (red) and DAPI (blue). Scale bar: 100 μm. (**k**) Graph shows the percentage of GFAP^+^ cells in DAPI^+^ cells (n = 4, biologically independent experiments, F (3,12) = 2.108, df = 12, *p* = 0.1525 for main effect of group, assessed by one-way ANOVA). (**l**) Representative images show immunocytochemistry for Nestin (green), Ki67 (red) and DAPI (blue). Scale bar: 100 μm. (**m**) Graph shows the percentage of Nestin^+^ cells in DAPI^+^ cells (n = 4, biologically independent experiments, F (3,12) = 0.7736, df = 12, *p* = 0.5308 for main effect of group, assessed by one-way ANOVA). (**n**) Graph shows the percentage of Ki67^+^ cells in Nestin^+^ cells (n = 4, biologically independent experiments, F (3,12) = 0.8434, df = 12, *p* = 0.4961 for main the effect of group, assessed by one-way ANOVA). (**o**) Representative images show immunocytochemistry for PDGFRα (green), Ki67 (red) and DAPI (blue). Scale bar: 100 μm. (**p**) Graph shows the percentage of PDGFRα^+^ cells in DAPI^+^ cells (n = 4, biologically independent experiments, F (3,12) = 6.297, df = 12, *p* = 0.0082 for main effect of group, *p* = 0.0221 for w/o microglia vs. scramble shRNA, assessed by one-way ANOVA, followed by Tukey–Kramer tests). (**q**) Graph shows the percentage of Ki67^+^ cells in PDGFRα^+^ cells (n = 4, biologically independent experiments, F (3,12) = 5.052, df = 12, *p* = 0.0172 for main effect of group, *p* = 0.018 for w/o microglia vs. scramble shRNA, assessed by one-way ANOVA, followed by Tukey–Kramer tests). *NS* not significant, *p < 0.05, **p < 0.01, ***p < 0.001. Error bars represent mean ± SEM.

To assess the sustainable effects of ASD-related genes on microglia, we used lentivirus encoding short hairpin RNA (shRNA) against Chd8 or Tsc2 (Fig. 4a). We confirmed the reduction of Chd8 and Tsc2 expression by quantitative RT-PCR after 7 days of infection (Fig. 4b,c). We then prepared NSCs obtained from neurospheres prepared from the P1 subventricular zone (SVZ), where oligodendrocytes are generated^32,33^ and developed during the neonatal period. We cultured lentivirus-infected microglia co-cultured with NSCs from the SVZ and counted the number of MBP^+^ oligodendrocytes in DAPI^+^ cells. The number of MBP^+^ oligodendrocytes was reduced in the presence of microglia infected with lentivirus encoding either Chd8 or Tsc2 shRNA compared with microglia infected with scramble shRNA coding lentivirus (Fig. 4d,e). Moreover, we stained with myelin-associated glycoprotein (MAG), which starts to be expressed before MBP in differentiating cultures. The number of MAG^+^ cells was also reduced in the presence of microglia infected with lentivirus encoding either Chd8 or Tsc2 shRNA (Fig. 4f,g). These results support our finding that Chd8 and Tsc2 play a role in microglia-mediated oligodendrocyte differentiation.

Oligodendrocyte differentiation was also regulated by other neural cells^34^. Therefore, it is possible to suppose that Chd8 or Tsc2 regulates microglia-mediated oligodendrocyte differentiation through other cells. To determine the possibility, we observed the number of neurons, astrocytes, NPCs, and OPC after co-culturing NSCs and microglia infected with lentivirus. The number of βIII-tubulin^+^ neurons or GFAP^+^ astrocytes was not affected by microglia (Fig. 4h–k). The number of Nestin^+^ NPC and Ki67^+^ cells in Nestin^+^ NPCs was not affected by microglia (Fig. 4l–n). These results suggest that Chd8 or Tsc2 in microglia do not affect NPC proliferation and differentiation of neurons or astrocytes. Although the number of PDGFRα^+^ OPC and the percentage of Ki67^+^ cells in PDGFRα^+^ OPC was increased in the presence of microglia, Chd8 or Tsc2 knockdown in microglia did not affect the number of OPC, and Ki67^+^ proliferating OPC compared with microglia infected with scramble shRNA- coding lentivirus (Fig. 4o–q), suggesting that microglia regulate oligodendrocyte differentiation in Chd8 or Tsc2-dependent manner without affecting neurons, astrocytes, NPC, and OPC.

### Chd8 or Tsc2 regulates numerous gene expression in microglia

**Figure 5:**
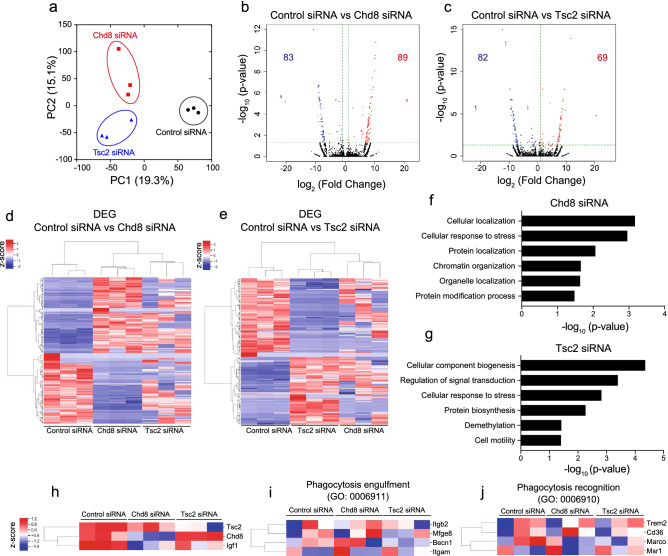
Chd8 or Tsc2 regulates numerous gene expression and biological processes in primary microglia. (**a**) PCA analysis showing gene expression variance from control siRNA (black, n = 3), Chd8 siRNA (red, n = 3), Tsc2 siRNA (blue, n = 3)-treated microglia. Percentages represent variance obtained by each principal component. (**b**) Volcano plot showing differentially expressed genes (DEGs) between control siRNA-treated microglia and Chd8 siRNA-treated microglia. (**c**) Volcano plot showing DEGs between control siRNA-treated microglia and Tsc2 siRNA-treated microglia. (**d**) Heatmap showing the expression of DEGs found between control siRNA and Chd8 siRNA-treated microglia. (**e**) Heatmap showing the expression of DEGs found between control siRNA and Tsc2 siRNA-treated microglia. (**f**) Graph showing the − log (*p*-value) and GO terms enriched in the DEGs found between control siRNA and Chd8 siRNA. (**g**) Graph showing the − log (*p*-value) and GO terms enriched in the DEGs found between control siRNA and Tsc2 siRNA. (**h**) Heatmap showing Tsc2, Chd8, and Igf1 expression in each siRNA treated microglia. (**i**) Heatmap showing the expression of phagocytosis engulfment-related genes. (**j**) Heatmap showing the expression of phagocytosis recognition-related genes.

To investigate the molecular profiles of microglia with Chd8 or Tsc2 deficiency, we performed RNA sequencing (RNA-seq) in primary microglia treated with control, Chd8, or Tsc2 siRNA after LPS stimulation for three days. Principal component analysis (PCA) showed the difference of transcriptome among control siRNA, Chd8 siRNA, and Tsc2 siRNA treatment microglia (Fig. 5a). We analyzed differentially expressed genes (DEGs) using volcano plots and found that the expression of 83 genes was significantly reduced and 89 genes were increased in Chd8 siRNA-treated microglia (Fig. 5b,d). Alternatively, the expression of 82 genes was significantly reduced, and 69 genes were increased in Tsc2 siRNA-treated microglia (Fig. 5c,e). To investigate the properties of DEGs, we classified 172 genes (for vs. Chd8 siRNA) and 151 genes (vs. Tsc2 siRNA) by Gene Ontology (GO) analysis of the biological process. Numerous GO terms such as “Cellular localization”, “Cellular response to stress”, “Protein localization”, “Chromatin organization”, “Organelle localization”, and “Protein modification process” were rich in DEGs of Chd8 siRNA (Fig. 5f). Alternatively, GO terms “Cellular component biogenesis”, “Regulation of signal transduction”, “Cellular response to stress”, “Protein biogenesis”, “Demethylation”, and “Cell motility” were rich in DEGs of Tsc2 siRNA (Fig. 5g). From these results, both Chd8 and Tsc2 regulate numerous biological processes, especially protein synthesis and localization. We also confirmed the reduction of Tsc2 or Chd8 expression in Tsc2 siRNA-or Chd8 siRNA treated microglia, respectively (Fig. 5h). Moreover, Igf1 expression was also reduced in Chd8 or Tsc2 siRNA treatment (Fig. 5h). These results suggest that either Chd8 or Tsc2 contributes to the expression of genes related to numerous biological processes including Igf1.

Tsc2 deficiency impairs phagocytosis activity in microglia. To determine whether Tsc2 regulates the expression of phagocytosis-related genes, we analyzed the expression of Itgam, Becn1, Itgb2, and Mfge8, which are annotated with phagocytosis engulfment. However, the expression of these genes was not significantly different under Tsc2 siRNA treatment, suggesting that impairment of phagocytosis activity during Tsc2 deficiency was not due to changes in the expression of phagocytosis-related genes (Fig. 5i). Microglia play a role in the engulfment of excessive myelin and synapses^35^. We also analyzed the expression of synapse- or myelin recognition-related genes, such as Msr1, Marco, Cd36, and Trem2. However, there were no significant differences between the groups (Fig. 5j). These results indicate that Tsc2-regulated phagocytosis activity is independent of the expression change of genes that are well-established for phagocytosis recognition.

### Chd8 or Tsc2 deficiency in microglia impaired oligodendrogenesis in vivo

**Figure 6:**
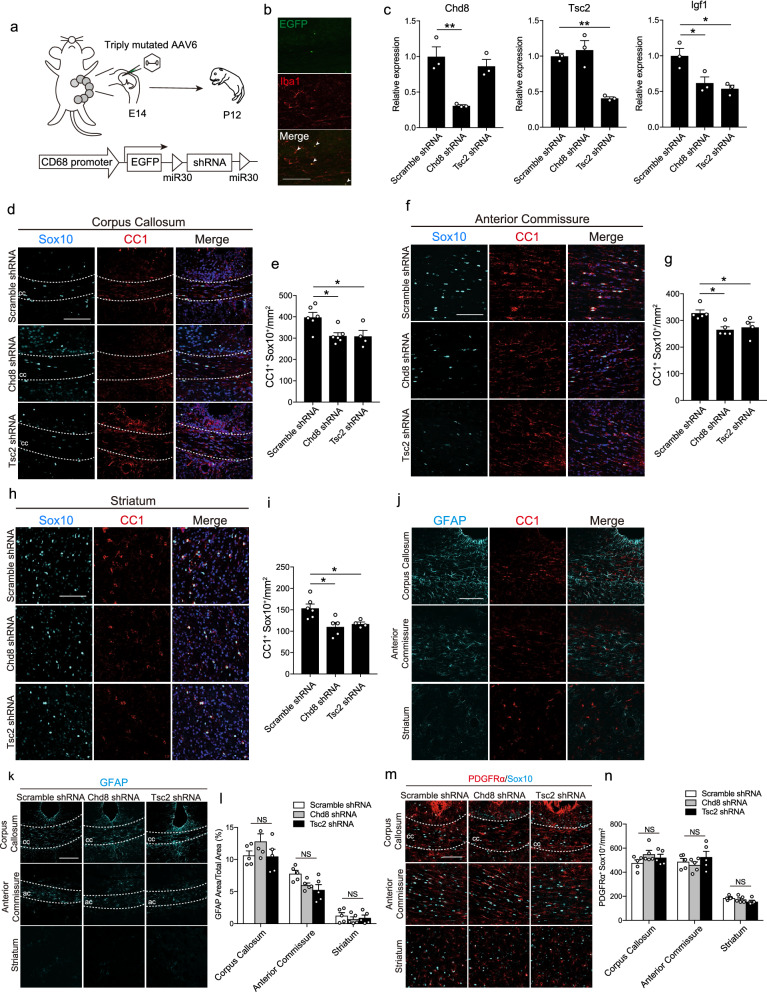
Chd8 or Tsc2 deficiency in microglia inhibits oligodendrocyte development in vivo. (**a**) Triply mutated AAV6 particles encoding EGFP and miR-30-based shRNA under control of the CD68 promoter were intraventrically injected to embryonic mice brain at E14. The following analyses was performed at P12. (**b**) Representative immunohistochemistry images for EGFP (green) and Iba1 (red) at P12. White arrowheads represent EGFP^+^ microglia. Scale bar: 100 μm. (**c**) Graph showing the mRNA expression level of Chd8, Tsc2, and Igf1 in EGFP^+^ CD45^mid^ CD11b^+^ microglia purified from the brain of P12 neonatal mice by flow cytometry (Scramble shRNA: n = 3; Chd8 shRNA: n = 3; Tsc2 shRNA: n = 3, biologically independent experiments, Chd8: F (2, 6) = 14.66, df = 6, *p* = 0.0049 for the main effect of the group, *p* = 0.0053 for scramble shRNA vs Chd8 shRNA, Tsc2: F (2, 6) = 21.43, df = 6, *p* = 0.0019 for main effect of group, *p* = 0.0046 for scramble shRNA vs Tsc2 shRNA, Igf1: F (2, 6) = 9.084, df = 6, *p* = 0.0153 for main effect of group, *p* = 0.0374 for scramble shRNA vs Chd8 shRNA, *p* = 0.0171 for scramble shRNA vs Tsc2 shRNA, assessed by one-way ANOVA, followed by Tukey–Kramer tests). (**d**) Representative immunohistochemical images for Sox10 (cyan), CC1 (red) and DAPI (blue) in the corpus callosum (cc). Scale bar: 100 μm. (**e**) Graph of the number of CC1^+^ Sox10^+^ oligodendrocytes in the corpus callosum (Scramble shRNA: n = 6; Chd8 shRNA: n = 6; Tsc2 shRNA: n = 4, biologically independent experiments, F (2, 13) = 6.305, df = 13, *p* = 0.0122 for main effect of group, *p* = 0.0195 for scramble shRNA vs. Chd8 shRNA, *p* = 0.0329 for scramble shRNA vs. Tsc2 shRNA, assessed by one-way ANOVA, followed by Tukey–Kramer tests). (**f**) Immunohistochemical images for Sox10 (cyan), CC1 (red) and DAPI (blue) in the anterior commissure. Scale bar: 100 μm. (**g**) Graph of the number of CC1^+^ Sox10^+^ oligodendrocytes in the anterior commissure (Scramble shRNA: n = 5; Chd8 shRNA: n = 5; Tsc2 shRNA: n = 4, biologically independent experiments, F (2, 11) = 6.818, df = 11, *p* = 0.0119 for main effect of group, *p* = 0.0138 for scramble shRNA vs. Chd8 shRNA, *p* = 0.0431 for scramble shRNA vs. Tsc2 shRNA, assessed by one-way ANOVA, followed by Tukey–Kramer tests). (**h**) Immunohistochemical images for Sox10 (cyan), CC1 (red) and DAPI (blue) in the striatum. Scale bar: 100 μm. (**i**) Graph of the number of CC1^+^ Sox10^+^ oligodendrocytes in the striatum (Scramble shRNA: n = 6; Chd8 shRNA: n = 5; Tsc2 shRNA: n = 4, biologically independent experiments, F (2, 11) = 7.296, df = 12, *p* = 0.0084 for main effect of group, *p* = 0.0105 for scramble shRNA vs. Chd8 shRNA, *p* = 0.0390 for scramble shRNA vs. Tsc2 shRNA, assessed by one-way ANOVA, followed by Tukey–Kramer tests). (**j**) Immunohistochemistry for GFAP (cyan) and CC1 (red) in the corpus callosum, anterior commissure, and striatum. Scale bar: 200 μm. (**k**) Immunohistochemistry for GFAP (cyan) in the corpus callosum (cc), anterior commissure (ac) and striatum in the brain of scramble, Chd8 and Tsc2 shRNA-treated mice. Scale bar: 100 μm. (**l**) Graph of the area of GFAP^+^ astrocytes in the corpus callosum, anterior commissure, and striatum (Scramble shRNA: n = 5; Chd8 shRNA: n = 5; Tsc2 shRNA: n = 5, biologically independent experiments, F (2, 12) = 0.9855, df = 2, *p* = 0.4015 for main effect of group, assessed by two-way ANOVA). (**m**) Immunohistochemistry for PDGFRα (red) and Sox10 (cyan) in the corpus callosum (cc), anterior commissure, and striatum. Scale bar: 100 μm. (**n**) Graph showing the number of PDGFRα^+^ Sox10^+^ cells in the corpus callosum (cc), anterior commissure and striatum in the brain of scramble, Chd8 and Tsc2 shRNA-treated mice (Scramble shRNA: n = 5; Chd8 shRNA: n = 5; Tsc2 shRNA: n = 5, biologically independent experiments, F (2, 12) = 0.5092, df = 2, *p* = 0.6134 for main effect of group, assessed by two-way ANOVA). *NS* not significant, *p < 0.05. **p < 0.01. Error bars represent mean ± SEM.

To examine the effect of Chd8 or Tsc2 deficiency in microglia on oligodendrocyte differentiation in vivo, we used AAV6, which has triply mutated capsid variants (mAAV6)^36^ to reduce Chd8 or Tsc2 expression in microglia. We inserted enhanced green fluorescent protein (EGFP) and miR-30-based shRNA into the AAV construct under the CD68 promoter to enhance specificity for microglia (Fig. 6a). At E14, we injected mAAV6 into the embryo lateral ventricle, and performed histological analysis at P12. We confirmed EGFP fluorescence in ionized calcium binding adapter protein 1 (Iba1)^+^ microglia in the corpus callosum (Fig. 6b). Additionally, we purified EGFP^+^ microglia from P12 brain and found that the expression levels of Chd8, Tsc2, and Igf1 in microglia were reduced in Chd8 or Tsc2 shRNA treatment, which was consistent with in vitro studies (Fig. 6c).

To label oligodendrocytes sparsely by immunohistochemistry, we used CC1 monoclonal antibody that recognizes adenomatous polyposis coli (APC). As CC1 monoclonal antibody also recognizes Quaking 7 protein^37^, which is expressed in oligodendrocytes and some astrocytes, we defined oligodendrocytes as CC1 and Sox10 (specific marker of oligodendrocyte-lineage cells) double-positive cells. We counted the number of CC1^+^ Sox10^+^ oligodendrocytes and found that a low number of CC1^+^ Sox10^+^ oligodendrocytes were detected in the corpus callosum (Fig. 6d,e), anterior commissure (Fig. 6f,g), and striatum (Fig. 6h,i) of mice treated with Chd8 or Tsc2 shRNA compared with control (scramble shRNA treated) mice. To examine the possibility that the reduction of oligodendrocyte number by Chd8 or Tsc2 shRNA was due to the oligodendrocyte death, we observed Cleaved Caspase-3^+^ Olig2^+^ double positive cells (apoptotic oligodendrocyte-lineage cells) in the Chd8 or Tsc2 shRNA-treated brain. However, there were few Cleaved Caspase-3^+^ Olig2^+^ double positive cells even in Chd8 or Tsc2 shRNA-treated brain (Supplementary Fig. S1a). In addition, there was no significant difference of Olig2^+^cell number between the groups (Supplementary Fig. S1b), indicating that our observation about the reduction of oligodendrocytes does not depend on the difference of oligodendrocyte survival. Alternatively, CC1^+^ GFAP^+^ cells were not found in these regions (Fig. 6j). GFAP^+^ area (Fig. 6k,l), and the number of PDGFRα^+^ Sox10^+^ OPC (Fig. 6m,n) was not affected by Chd8 or Tsc2 shRNA treatment in the corpus callosum, anterior commissure, and striatum. These results demonstrate that microglial Chd8 or Tsc2 contribute to oligodendrocyte development in vivo without affecting astrocytes or OPC.

## Discussion

Microglia are key players in causing structural abnormalities in the brain during development, and they play a key role in the pathogenesis of neuronal disorders. Regarding the involvement of ASD pathology, microglia engulf synapses of neurons undergoing synapse formation during brain development^17,30^, and this developmental microglial dysfunction causes abnormal behavioral phenotype such as social interaction deficits^38,39^. Considering the presence of ASD-related gene expression in microglia, in this study, we asked whether microglial ASD-associated gene expression is autonomously involved in microglial functions of the cell.

According to our qPCR and RNAseq analyses, Igf1 expression was reduced by silencing Chd8 or Tsc2 in primary microglia. During normal development, a subset of microglia located in the white matter promotes myelination by secreting Igf1^16^ in a phosphoinositide 3-kinase (PI3K)-Akt-dependent manner^40^. Our cell culture experiments revealed that microglia with Chd8 or Tsc2 knocked down reduced the differentiation efficiency of oligodendrocytes, which is in the same direction as the decrease in Igf1 expression in microglia regulated by ASD-related genes. It has been reported that Igf1 expression is regulated by several transcriptional factors such as CCAAT-enhancer binding protein β (C/EBPβ), C/EBPγ, Interferon regulatory factor 8, and Jun^41,42^. However, our RNAseq analysis could not detect significant difference of these gene expressions in Chd8 or Tsc2 deficient microglia (data not shown). It has also been reported that Chd8 interacts with C/EBPβ and promotes its transactivation activity^43^. In addition, the Tsc1/Tsc2 complex inhibits mTORC1 activity, preventing C/EBPβ translocation^44^. The reduction of Igf1 expression by Chd8 and Tsc2 silencing may be mediated by C/EBPβ inactivation without change of mRNA level of C/EBPβ.

We found that Tsc2 deficiency impaired phagocytosis activity in primary microglia. Tsc2 forms a TSC complex with Tsc1, which inhibits mTOR signaling^10^. Knocking out microglial Tsc1 increases phagocytosis activity^45^. Consistent with this finding, mTOR deletion in microglia led to the impairment of phagocytosis both in vitro and in vivo^46^. In contrast, Tsc2^+/–^ mice showed impaired autophagic activity^47^, and our study showed Tsc2-deficient microglia had reduced phagocytosis activity. This discrepancy may be explained by the difference in signal transduction between Tsc1 and Tsc2. One of the well-established differences between Tsc1 and Tsc2 is the function of Tsc1 in Smad2/3 phosphorylation in the presence of TGF-β, which controls epithelial-to-mesenchymal transition^48^. However, in microglia, TGF-β and Smad3 activation increases phagocytosis activity^49^; therefore, TSC-mediated intracellular signaling may have cell-type specific regulation. Further studies on the intracellular mechanism will contribute to the understanding of Tsc2-mediated intracellular signaling that regulates phagocytosis.

In this study, we found a decrease in the number of oligodendrocytes generated from NSCs or OPCs co-cultured with Chd8- or Tsc2-deficient microglia. However, we did detect that Tsc2-deficient microglia showed altered phagocytosis activity. As we used transwells for co-culture experiments, the decrease in the number of oligodendrocytes that we observed does not depend on the phagocytic function of microglia in vitro. However, we should note that the recent study showed that microglia regulate myelination by engulfment of OPC in developing brain^19^. It is generally accepted that microglia eliminate synapses and myelin during brain development in a cell–cell contact-dependent manner. Molecules involved in phagocytosis of synapses and myelin by microglia included complement receptor-3 (Itgam), scavenger receptor (Msr1, Marco, Cd36), and Trem2^50^. Although our RNAseq analysis showed that Chd8 or Tsc2 deficiency did not affect the expression of the well-established genes which is associated with the phagocytosis of synapses and myelin, there is still the possibility that ASD-related microglia contribute to phagocytosis of synapses and myelin because the expression of some of the above molecules is dramatically regulated by several conditions (such as developmental regulation of Trem2)^51^.

Previous studies have demonstrated that myelin dysfunction was observed in ASD patients^4^, and genetic analysis showed that myelin development-related genes were associated with ASD^52,53^. Therefore, dysfunctional myelin development may be key to understanding ASD pathology. Regarding Chd8 and Tsc2, oligodendrocyte-specific loss of function experiments showed the impairment of oligodendrocyte differentiation and causes hypomyelination through the epigenetic modulation of gene expression, which is involved in oligodendrocyte proliferation and myelination^8,9,13^. In this study, we focused on the expression of ASD-related genes in microglia and revealed a novel function of ASD-related genes in myelin pathology. However, we should note that the expression of ASD-related genes is not limited to the brain, but is also detected systemically. Recently, we found that changes in systemic environments control myelin development directly via circulating factors^54^, providing more opportunities to understand the contribution of ASD-related genes to neuropathology.

## Materials and methods

### Animals

Pregnant mice at embryonic day (E) 14 and E18 of C57BL/6J mice were purchased from SLC Japan. All experiments procedures were approved by the Animal Care Committee of National Center of Neurology and Psychiatry (2018034R9). The mice were housed in an air-conditioned room at 23 ± 1 °C with a 12-h light–dark cycle under specific pathogen-free conditions and had free access to water and food. The mice were randomly assigned to groups. All experiments were conducted according to the relevant guidelines and regulations including Animal Research Reporting of In Vivo Experiments (ARRIVE) guidelines.

### Microglia and OPC primary culture

Whole brains were dissected from E18 mice, and incubated with 0.25% trypsin (Gibco) in Dulbecco's Modified Eagle Medium (DMEM; Gibco) for 15 min at 37 °C, followed by treatment with DNase I (Sigma Aldrich) for 1 min at 37 °C. Cell suspension was washed by DMEM containing 10% fetal bovine serum (FBS) and centrifuged at 450×*g* for 10 min. The isolated cells were resuspended with DMEM containing 10% FBS, and filtered with 70 μm nylon cell strainer. Cells were plated on poly-l-lysine precoated 75 cm^2^ tissue culture flask (IWAKI) in DMEM supplemented with 10% FBS and penicillin–streptomycin (Thermo Fisher Scientific). After 12–15 days, culture flasks were gently shaking for 30 min, and supernatant was centrifuged at 1500 rpm for 10 min. The cell pellet was resuspended with microglia culture medium supplemented with 50% of the supernatant, 10% FBS in DMEM, and plated to 96 well plate. Each biological replicate represents a culture prepared from a distinct mouse brain. All experiments were repeated at least three times.

For primary OPCs, mixed glial cells with removed microglia were detached from the culture flask by treatment with 0.05% trypsin for 5 min and replated to fresh culture flasks. After 30 min of incubation, the culture medium was centrifuged at 500×*g*, and the cell pellet was resuspended in OPC culture medium supplemented with sodium pyruvate (1:100, Gibco), apo-transferrin (50 μg/mL, Sigma-Aldrich), bovine serum albumin (0.1%, Sigma-Aldrich), insulin (5 μg/mL, Sigma-Aldrich), biotin (10 nM, Sigma-Aldrich), hydrocortisone (10 nM, Sigma-Aldrich), sodium selenite (30 nM, Sigma-Aldrich), and penicillin–streptomycin (1:100, Life Technologies). OPCs were plated onto poly-l-lysine-coated 96 well plates.

### siRNA treatment for microglia

Primary microglia were transduced with Lipofectamine RNAiMAX Reagent (Thermo Fisher Scientific) according to the manufacture’s protocol. Briefly, 0.5 pmol of siRNA for BLOCK-it Alexa Fluor Red Fluorescent control (14750100: Thermo Fisher Scientific), negative control (4390843: Thermo Fisher Scientific), Chd8 (4390771: Thermo Fisher Scientific), and Tsc2 (AM16708: Thermo Fisher Scientific) were diluted with OPTI-MEM (Invitrogen), and mixed with 0.15 μL of RNAiMAX Reagent, which were diluted with OPTI-MEM. Complex was added to primary microglia plated on 96 well plate. After 4 h, culture medium was changed to new microglia culture medium.

### Lentivirus production

The scramble shRNA plasmid was a gift from David Sabatini (Addgene plasmid #1864, http://www.addgene.org/1864/; RRID:Addgene_1864). To generate the shRNA construct, the scramble sequence was digested with EcoRI and AgeI, and shRNA sequences against Chd8 and Tsc2 were inserted with the DNA Ligation Kit Ver 2.1 (Takara). The shRNA target sequences of Chd8 and Tsc2 were TCGGGAAGCTTGCCATATTAT and CCTCTCTCTTAAACTTGATA, respectively. pLenti, pCAG-HIVgp, and p-CMV-VSV-G plasmids were transfected into Lenti-X 293 T cells (Takara) with polyethylenimine (PEI) Max (Polysciences). After 3 days of transfection, the supernatant was centrifuged at 1000×*g* for 10 min, and filtered with 0.45 μm filter. Then, the supernatant was centrifuged at 50,000×*g* for 2 h (SW28, Beckman). The pellet was resuspended in PBS and concentrated with an Amicon Ultra-15 (Millipore). pCAG-HIVgp and p-CMV-VSV-G plasmids were generated by Dr. Hiroyuki Miyoshi and purchased from RIKEN Bioresource Research Center.

### AAV infection of microglia

pAAV plasmids encoding EGFP and miR-30-based shRNA under the CD68 promoter were prepared by VectorBuilder. The shRNA sequences were as follows: scramble shRNA: ccctaaggttaagtcgccctcgtagtgaagccacagatgtacgagggcgacttaaccttaggt; Chd8 shRNA: atgaacgtattgatgggcgagttagtgaagccacagatgtaactcgcccatcaatacgttcag; Tsc2 shRNA: cttgacgaatacattgcatcaatagtgaagccacagatgtattgatgcaatgtattcgtcaat. The modified AAV6 capsid (Y731F/Y705F/T492V) was a kind gift from Todd E. Golde (University of Florida Board of Trustees)^36^. pAAV, pHelper, and modified AAV6 capsids were transfected to AAV293 cells (Takara) by PEI Max. After 3 days, AAV was purified with an AAVpro Purification kit (Takara) and concentrated with an Amicon Ultra-15 (Millipore). To inject AAV into the mouse brain, E14 pregnant mice were anesthetized with isoflurane (3%), and 1 μL of AAV containing 0.01% fast green was injected into the lateral ventricle of the embryonic mouse brain, and P12 neonatal mice were analyzed.

### Flow cytometry

Primary microglia, which were transduced fluorescent-labeled siRNA, were treated with APC conjugated anti-CD11b (1:200; Biolegend) on ice for 30 min. After three times wash, primary microglia were carried out flow cytometry analysis by FACSCantoII fluidics system (BD Biosciences), and the data were analyzed with FlowJo software (Tree Star).

### Phagocytosis assay

Three days after siRNA transduction, microglia phagocytic activities were measured with Phagocytosis Assay Kit (Cayman). Microglia were incubated for 24 h with latex beads coated with fluorescently-labeled rabbit IgG, followed by 1 min incubation with trypan blue. Microglia were fixed with 4% paraformaldehyde and permeabilized with PBS containing 0.1% Triton X-100 for 15 min. Microglia were incubated with Isolectin B4 Conjugates (10 μg/mL: Invitrogen) in PBS containing 0.1% Triton X-100 for 2 h followed by three times PBS washing. Counterstaining were carried out with DAPI (1:2000, DOJIDO). Images were acquired using a confocal laser scanning microscope (FV3000, Olympus).

### Co-culture of microglia and NSCs

For generating neurospheres, telencephalons were dissected from E14 or P1 of C57BL/6J mice and the tissue was dispersed into single cells by triturating several times. Cells were plated on 100 mm dish (CELLSTAR) at 1.0 × 10^6^ cells/dish in DMEM/Nutrient Mixture F-12 Ham medium (DMEM/F12; Sigma Aldrich) supplemented with B27 supplement (1:200; Thermo Fisher Scientific), Penicillin–Streptomycin (1:100; Thermo Fisher Scientific), Epidermal Growth Factor (EGF, 20 ng/mL; Peprotech) and basic Fibroblast Growth Factor (bFGF, 20 ng/mL; PEPROTECH) and cultured for 7 days.

For co-culturing microglia and NSCs, neurospheres were incubated with 0.25% trypsin (Gibco) in DMEM/F12 for 3 min at 37 °C, and dissociated NSCs were plated on poly-ornithine (30 μg/mL)-precoated 96 well plate (Corning) at a density of 2 × 10^4^ cells/well in DMEM/F12 supplemented with Penicillin–Streptomycin (1:100). Control siRNA, Chd8 siRNA, and Tsc2 siRNA treated microglia were activated with LPS (10 ng/mL) for 6 h and washed carefully to remove residual LPS. Microglia were not stimulated by LPS in the experiments shown in Figs. 1b,c–e, 4b,c. For lentivirus infection, microglia were cultured in the presence of lentivirus and polybrene (8 μg/mL; Nacalai Tesque) for 24 h. Culture media containing lentivirus were replaced with fresh media, and microglia were incubated for an additional 2 days. Microglia were plated on 96 well Transwell plates (pore size 0.4 μm; Corning), which were plated on NSCs at a density of 2.0 × 10^4^ cells/well. NSCs and microglia were co-cultured for 7 days, and immunocytochemistry was performed.

### Immunocytochemistry

Cells were fixed with 4% paraformaldehyde (PFA; Merck) at room temperature for 30 min and permeabilized with PBS containing 0.1% Triton X-100 for 15 min. Then, cells were blocked with 3% normal donkey serum (Sigma-Aldrich) for 1 h at room temperature and treated with primary antibody overnight at 4 °C. Primary antibodies were used as follows; rabbit anti-βIII-Tubulin (1:2000, PRB-435P; Biolegend), rat anti-MBP (1:500, ab7349; Abcam), rabbit anti-MAG (1:500, 9043; Cell Signaling Technology), mouse anti-GFAP (1:1000, GA-5, Sigma Aldrich), rat anti-GFAP (1:500, 2.2B10; Thermo Fisher Scientific), goat anti-PDGFRα (1:1000, AF1062; R&D Systems), rabbit anti-Ki67 (1:500, ab16667; Abcam), mouse anti-Olig2 (1:1000, 211F1.1; Millipore), rat anti-Nestin (1:1000, rat-401; Millipore). After three times wash, cells were incubated with secondary antibodies at room temperature for 1 h. Secondary antibodies were used as follows, Alexa 488-conjugated donkey anti-mouse IgG, Alexa 594-conjugated donkey anti-rat IgG (1:500; Thermo Fisher Scientific) and DAPI (1:2000; DOJINDO). After three times wash, cells were observed with IN Cell Analyzer 2000 (GE Healthcare) or confocal laser scanning microscope (FV3000, Olympus).

### Immunohistochemistry

Mice were anesthetized with a mixture of medetomidine hydrochloride (0.3 mg/kg), midazolam (4 mg/kg), and butorphanol (5 mg/kg). Mice were transcardially perfused with ice-cold PBS then 4% PFA (Merck). Dissected brains were immersed in 4% PFA overnight at 4 °C, followed by 30% sucrose (pH 7.2) overnight at 4 °C. Brains were embedded in optimal cutting temperature compound (Tissue-Tek) and cut to a thickness of 30 μm on a cryostat (Leica). For immunohistochemistry, sections were treated with PBS containing 0.1% Triton X-100 for 10 min twice, and blocked with 3% normal donkey serum at room temperature for 1 h. Sections were treated with primary antibodies: rabbit anti-Iba1 (1:3000, 019-19741; Wako), rat anti-GFP (1:500, 04404-84; Nacalai Tesque), mouse CC1 monoclonal antibody (1:500, OP80; Calbiochem), rabbit anti-Sox10 (1:1000, ab155279; Abcam), rabbit anti-GFAP (1:1000, ab207165; Abcam), goat anti-PDGFRα (1:1000, AF1062; R&D Systems), goat anti-Olig2 (1:1000, AF2418; R&D Systems), rabbit anti-Cleaved Caspase-3 (1:500, 9661; Cell Signaling Technology) overnight at 4 °C. After three washes, sections were treated with secondary antibodies: Alexa Fluor 488-conjugated donkey anti-rat IgG, Alexa Fluor 568-conjugated donkey anti-rabbit IgG, Alexa Fluor 568-conjugated donkey anti-mouse IgG (all: 1:500; Thermo Fisher Scientific), and DAPI (1:2000; DOJINDO), and sections were mounted with ProLong Glass Antifade Mountant (Thermo Fisher Scientific). Images were acquired using a confocal microscope (FV3000, Olympus). GFAP area was calculated by dividing the GFAP-positive area in the area of the white matter (corpus callosum and anterior commissure) or total area (striatum).

### RNA-seq

After 3 days of treatment with siRNA against primary microglia, RNA was isolated using TRIzol Reagent (Invitrogen). RNA quality was checked with an Agilent Bioanalyzer and RNA with an RNA integrity number > 8 were analyzed. Libraries were generated using Illumina standard Total RNA Prep and sequenced using Novaseq 6000 (Illumina) at a depth of 20 million 150 bp paired-end reads. Read counts and TPM were calculated with salmon (v.0.14.1) on the mm10 mouse genome reference and Tximport package (v1.14.2) on R (v3.6.3). Normalization was performed with the DESeq2 (v 1.26.0), and DEGs were defined by a false discovery rate (FDR) < 0.05 and fold-change > 1.5. PCA was conducted by scikit-learn module (v0.22.2. post1) on Python (v3.7.1). GO analysis was performed using the Database for Annotation, Visualization, and Integrated Discovery (DAVID, v.6.8)^55,56^.

### Quantitative RT-PCR

Six hours after LPS treatment, microglia RNA was extracted with TRIzol Reagent (Invitrogen) and cDNA was synthesized using High Capacity cDNA Reverse Transcription Kit (Applied biosystems). Quantitative RT-PCR was conducted with KAPA SYBR FAST qPCR Master Mix (Kapa Biosystems) or KAPA PROBE Fast qPCR kit (Kapa Biosystems). PCR reactions were analyzed with CFX Connect Real-time PCR Detection System (Bio-rad). Gene expressions were calculated by ΔΔCt method. Sequences of PCR primers are as follows: Chd8: 5′-cagaggaggagggtgaaaagaac-3 (forward) and 5′-gagttgtcagacgatgtgttacgc-3′ (reverse); Tsc2: 5′-aaagattccggcttgaaggag-3′ (forward) and 5′-gcattcaccactcagttctctc-3′ (reverse); Gapdh: 5′-aggtcggtgtgaacggatttg-3′ (forward) and 5′-tgtagaccatgtagttgaggtca-3′ (reverse); Actb: 5′-ggctgtattcccctccatcg-3′ (forward) and 5′-ccagttggtaacaatgccatgt-3′ (reverse). Probes used were as follows: Gapdh (Assay ID: Mm99999915_g1, ThermoFisher Scientific), Tnf (Assay ID: Mm00443258_m1, ThermoFisher Scientific), Spp1 (Assay ID: Mm00436767_m1, Thermo Fisher Scientific), Il1b (Assay ID: Mm00434228_m1, Thermo Fisher Scientific), and Igf1 (Assay ID:Mm00439560_m1, Thermo Fisher Scientific).

### Cell sorting of microglia

P12 mice were anesthetized by intraperitoneal administration of a cocktail of domitor (0.3 mg/kg), dormicum (4 mg/kg), and butorphanol (5 mg/kg). Mice were transcardially perfused with ice-cold PBS, and dissected brains were digested with collagenase D (1 mg/mL; Roche) containing 2.5 mM calcium chloride at 37 °C for 30 min. After trituration, digested tissues were resuspended with 30% percoll (GE Healthcare), and 70% percoll was layered underneath, followed by centrifugation at 2000 rpm for 30 min at room temperature. Cells were treated with APC conjugated anti-CD11b (1:200; Biolegend) and pacific blue conjugated anti-CD45 (1:200; Biolegend) on ice for 30 min. GFP^+^ CD11b^+^ CD45^mid^ microglia were sorted with cell sorter SH800 (Sony). Total RNA was purified from sorted microglia by Trizol Reagent (Thermo Fisher Scientific). Quantitative RT-PCR was conducted with Quant-X One-Step qRT-PCR TB green Kit (Takara) and CFX Connect Real-time PCR Detection System (Bio-rad). Sequences of PCR primers of Igf1 are as follows: 5′-gtgagccaaagacacaccca-3′ (forward) and 5′-acctctgattttccgagttgc-3′ (reverse).

### Statistics

The data are presented as mean ± standard error of the mean (SEM). Student’s *t*-tests, one-way analysis of variance (ANOVA) and Tukey–Kramer post-hoc tests, two-way ANOVA tests were performed with GraphPad Prism 7 (GraphPad). *p* < 0.05 was considered as significant.

## Supplementary Information

Supplementary Figure 1.

Supplementary Legends.

## Data Availability

RNA-seq data have been deposited in the NCBI Gene Expression Omnibus (GEO) under the accession number GSE172014.

## References

[1] Abrahams BS, Geschwind DH (2008). Advances in autism genetics: On the threshold of a new neurobiology. Nat. Rev. Genet..

[2] Courchesne E (2011). Neuron number and size in prefrontal cortex of children with autism. JAMA J. Am. Med. Assoc..

[3] Hutsler JJ, Zhang H (2010). Increased dendritic spine densities on cortical projection neurons in autism spectrum disorders. Brain Res..

[4] Deoni SCL (2015). White-matter relaxation time and myelin water fraction differences in young adults with autism. Psychol. Med..

[5] Barnard RA, Pomaville MB, O’Roak BJ (2015). Mutations and modeling of the chromatin remodeler CHD8 define an emerging autism etiology. Front. Neurosci..

[6] Katayama Y (2016). CHD8 haploinsufficiency results in autistic-like phenotypes in mice. Nature.

[7] Hoffmann A, Spengler D (2021). Chromatin remodeler CHD8 in autism and brain development. J. Clin. Med..

[8] Marie C (2018). Oligodendrocyte precursor survival and differentiation requires chromatin remodeling by Chd7 and Chd8. Proc. Natl. Acad. Sci. U. S. A..

[9] Kawamura A (2020). Oligodendrocyte dysfunction due to Chd8 mutation gives rise to behavioral deficits in mice. Hum. Mol. Genet..

[10] Inoki K, Li Y, Xu T, Guan KL (2003). Rheb GTpase is a direct target of TSC2 GAP activity and regulates mTOR signaling. Genes Dev..

[11] Hooshmandi M, Wong C, Khoutorsky A (2020). Dysregulation of translational control signaling in autism spectrum disorders. Cell. Signal..

[12] Sato A (2012). Rapamycin reverses impaired social interaction in mouse models of tuberous sclerosis complex. Nat. Commun..

[13] Carson RP (2015). Hypomyelination following deletion of Tsc2 in oligodendrocyte precursors. Ann. Clin. Transl. Neurol..

[14] Bohlen CJ, Friedman BA, Dejanovic B, Sheng M (2019). Microglia in brain development, homeostasis, and neurodegeneration. Annu. Rev. Genet..

[15] Shigemoto-Mogami Y, Hoshikawa K, Goldman JE, Sekino Y, Sato K (2014). Microglia enhance neurogenesis and oligodendrogenesis in the early postnatal subventricular zone. J. Neurosci..

[16] Wlodarczyk A (2017). A novel microglial subset plays a key role in myelinogenesis in developing brain. EMBO J..

[17] Cunningham CL, Martínez-Cerdeño V, Noctor SC (2013). Microglia regulate the number of neural precursor cells in the developing cerebral cortex. J. Neurosci..

[18] Arnò B (2014). Neural progenitor cells orchestrate microglia migration and positioning into the developing cortex. Nat. Commun..

[19] Nemes-Baran AD, White DR, DeSilva TM (2020). Fractalkine-dependent microglial pruning of viable oligodendrocyte progenitor cells regulates myelination. Cell Rep..

[20] Hughes AN, Appel B (2020). Microglia phagocytose myelin sheaths to modify developmental myelination. Nat. Neurosci..

[21] Liu Y, Aguzzi A (2020). NG2 glia are required for maintaining microglia homeostatic state. Glia.

[22] Kirby L (2019). Oligodendrocyte precursor cells present antigen and are cytotoxic targets in inflammatory demyelination. Nat. Commun..

[23] Domingues HS, Portugal CC, Socodato R, Relvas JB (2016). Oligodendrocyte, astrocyte, and microglia crosstalk in myelin development, damage, and repair. Front. Cell Dev. Biol..

[24] Kim HJ (2017). Deficient autophagy in microglia impairs synaptic pruning and causes social behavioral defects. Mol. Psychiatry.

[25] Xu ZX (2020). Elevated protein synthesis in microglia causes autism-like synaptic and behavioral aberrations. Nat. Commun..

[26] Kumar H, Sharma B (2016). Minocycline ameliorates prenatal valproic acid induced autistic behaviour, biochemistry and blood brain barrier impairments in rats. Brain Res..

[27] Zhao C (2018). Dual requirement of CHD8 for chromatin landscape establishment and histone methyltransferase recruitment to promote CNS myelination and repair. Dev. Cell.

[28] Chen Shang Y, Zhong Chong Z, Wang S, Maiese K (2013). Tuberous sclerosis protein 2 (TSC2) modulates CCN4 cytoprotection during apoptotic amyloid toxicity in microglia. Curr. Neurovasc. Res..

[29] Marinelli C (2015). Ligand engagement of Toll-like receptors regulates their expression in cortical microglia and astrocytes. J. Neuroinflamm..

[30] Paolicelli RC (2011). Synaptic pruning by microglia is necessary for normal brain development. Science (80-)..

[31] Richardson WD, Kessaris N, Pringle N (2006). Oligodendrocyte wars. Nat. Rev. Neurosci..

[32] Goldman SA, Kuypers NJ (2015). How to make an oligodendrocyte. Development.

[33] Moore L, Bain JM, Loh JM, Levison SW (2014). PDGF-responsive progenitors persist in the subventricular zone across the lifespan. ASN Neuro.

[34] Mayoral SR, Chan JR (2016). The environment rules: Spatiotemporal regulation of oligodendrocyte differentiation. Curr. Opin. Neurobiol..

[35] Wang K (2021). Central nervous system diseases related to pathological microglial phagocytosis. CNS Neurosci. Ther..

[36] Rosario AM (2016). Microglia-specific targeting by novel capsid-modified AAV6 vectors. Mol. Ther. Methods Clin. Dev..

[37] Bin JM, Harris SN, Kennedy TE (2016). The oligodendrocyte-specific antibody ‘CC1’ binds Quaking 7. J. Neurochem..

[38] Zhan Y (2014). Deficient neuron-microglia signaling results in impaired functional brain connectivity and social behavior. Nat. Neurosci..

[39] Kopec AM, Smith CJ, Ayre NR, Sweat SC, Bilbo SD (2018). Microglial dopamine receptor elimination defines sex-specific nucleus accumbens development and social behavior in adolescent rats. Nat. Commun..

[40] Palacios N, Sánchez-Franco F, Fernández M, Sánchez I, Cacicedo L (2005). Intracellular events mediating insulin-like growth factor I-induced oligodendrocyte development: Modulation by cyclic AMP. J. Neurochem..

[41] Oberbauer AM (2013). The regulation of IGF-1 gene transcription and splicing during development and aging. Front. Endocrinol. (Lausanne)..

[42] Tang Y (2015). CCAAT-enhancer binding protein (C/EBP) β regulates insulin-like growth factor (IGF) 1 expression in porcine liver during prenatal and postnatal development. Mol. Cell. Biochem..

[43] Kita Y (2018). The autism-related protein CHD8 cooperates with C/EBPβ to regulate adipogenesis. Cell Rep..

[44] Yang T (2016). TSC1 controls IL-1β expression in macrophages via mTORC1-dependent C/EBPβ pathway. Cell. Mol. Immunol..

[45] Zhao X (2018). Noninflammatory changes of microglia are sufficient to cause epilepsy. Cell Rep..

[46] Zhao XF (2020). Microglial mTOR is neuronal protective and antiepileptogenic in the pilocarpine model of temporal lobe epilepsy. J. Neurosci..

[47] Tang G (2014). Loss of mTOR-dependent macroautophagy causes autistic-like synaptic pruning deficits. Neuron.

[48] Thien A (2015). TSC1 activates TGF-β-Smad2/3 signaling in growth arrest and epithelial-to-mesenchymal transition. Dev. Cell.

[49] Tichauer JE (2014). Age-dependent changes on TGFβ1 Smad3 pathway modify the pattern of microglial cell activation. Brain. Behav. Immun..

[50] Szepesi Z, Manouchehrian O, Bachiller S, Deierborg T (2018). Bidirectional microglia–neuron communication in health and disease. Front. Cell. Neurosci..

[51] Thrash JC, Torbett BE, Carson MJ (2009). Developmental regulation of TREM2 and DAP12 expression in the murine CNS: Implications for nasu-hakola disease. Neurochem. Res..

[52] Phan BDN (2020). A myelin-related transcriptomic profile is shared by Pitt-Hopkins syndrome models and human autism spectrum disorder. Nat. Neurosci..

[53] Castelijns B (2020). Hominin-specific regulatory elements selectively emerged in oligodendrocytes and are disrupted in autism patients. Nat. Commun..

[54] Ito M (2021). Age-dependent decline in remyelination capacity is mediated by apelin–APJ signaling. Nat. Aging.

[55] Huang DW, Sherman BT, Lempicki RA (2009). Systematic and integrative analysis of large gene lists using DAVID bioinformatics resources. Nat. Protoc..

[56] Huang DW, Sherman BT, Lempicki RA (2009). Bioinformatics enrichment tools: Paths toward the comprehensive functional analysis of large gene lists. Nucleic Acids Res..

