# Supplementary figures and images for: Microglial ASD-related genes are involved in oligodendrocyte differentiation
Source: Sci Rep. 2021 Sep 8;11:17825. doi: 10.1038/s41598-021-97257-9 (PMC8426463; doi:10.1038/s41598-021-97257-9)

# Supplementary Figure 1

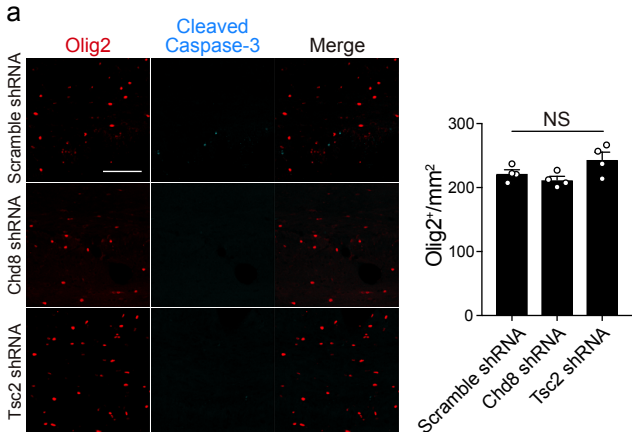

Supplement: Supplementary file 1 — Supplementary Figure 1. [file 41598_2021_97257_MOESM1_ESM.pdf]
