# Supplementary material for: Microglial ASD-related genes are involved in oligodendrocyte differentiation
Source: Sci Rep. 2021 Sep 8;11:17825. doi: 10.1038/s41598-021-97257-9 (PMC8426463; doi:10.1038/s41598-021-97257-9)
Supplement: Supplementary file 2 — Supplementary Legends. [file 41598_2021_97257_MOESM2_ESM.docx]

**Supplementary Figure 1. Chd8 or Tsc2 deficiency in microglia did not affect oligodendrocyte survival in vivo.** (a) Immunohistochemistry for Olig2 (red) and Cleaved Caspase-3 (cyan) in the P12 brain infected with mutated AAV6 encoding scramble, Chd8 or Tsc2 shRNA under CD68 promoter. Scale bar: 100 μm. (b) Graph showing the number of Olig2^+^ cells in the corpus callosum of scramble, Chd8 and Tsc2 shRNA-treated brain (Scramble shRNA: n = 4; Chd8 shRNA: n = 4; Tsc2 shRNA: n = 4, biologically independent experiments, F (2, 9) = 1.806, df = 9, *p* = 0.0611 for main effect of group, assessed by one-way ANOVA).
